# Altered Microbiome Promotes Pro-Inflammatory Pathways in Oesophago-Gastric Tumourigenesis

**DOI:** 10.3390/cancers16193426

**Published:** 2024-10-09

**Authors:** Nikhil Manish Patel, Pranav Harshad Patel, Ricky Harminder Bhogal, Kevin Joseph Harrington, Aran Singanayagam, Sacheen Kumar

**Affiliations:** 1 Department of Upper GI Surgery, The Royal Marsden NHS Foundation Trust, London SW3 6JJ, UK; nikhil.patel@rmh.nhs.uk (N.M.P.);; 2 The Upper Gastrointestinal Surgical Oncology Research Group, Division of Radiotherapy and Imaging, The Institute of Cancer Research, London SW7 3RP, UK; 3 Targeted Therapy Group, Division of Radiotherapy and Imaging, The Institute of Cancer Research, London SW7 3RP, UK; 4 Centre for Bacterial Resistance Biology, Department of Infectious Disease, Imperial College London, London SW7 2AZ, UK; 5 Department of Upper Gastrointestinal Surgery, Digestive Disease and Surgery Institute, Cleveland Clinic London Hospital, London SW1X 7HY, UK

**Keywords:** oesophago-gastric cancer, microbiome, inflammation, tumourigenesis, bacteria

## Abstract

**Simple Summary:**

Cancer of the upper digestive system is associated with poor survival due to difficulties in diagnosing the disease early, before it has spread around the body. Previous research has shown that the healthy bacteria within the body changes in response to medications, diet and infection. Some of these changes are associated with a greater risk of developing cancers of the oesophagus (gullet) and stomach. This occurs, at least in part, through inflammation. This process is the body’s natural response to infection, injury and exposure to potentially harmful substances, which, if uncontrolled can lead to diseases including cancer. This review article aims to explain the ways in which this happens. By developing a deeper understanding of these processes, advances in early diagnosis of this disease can be made, which may lead to better survival.

**Abstract:**

Introduction: The upper gastrointestinal microbiome is a dynamic entity that is involved in numerous processes including digestion, production of vitamins and protection against pathogens. Many external and intrinsic factors may cause changes in the proportions of bacteria within the microbial community, termed ‘dysbiosis’. A number of these have been identified as risk factors for a range of diseases, including oesophago-gastric carcinoma. Materials and Methods: A narrative review was conducted to elucidate the current evidence on the role of the microbiome in promoting oesophago-gastric tumourigenesis. Significant causes of dysbiosis including age, medications and GORD were examined and key pro-inflammatory pathways implicated in tumourigenesis and their interaction with the microbiome were described. Results and Discussion: An association between microbial dysbiosis and development of oesophago-gastric cancer may be mediated via activation of pro-inflammatory pathways, the inflammasome and the innate immune system. Advances in sequencing technology allow microbial communities to be fingerprinted by sequencing the 16S rRNA gene, enabling a deeper understanding of the genera that may be implicated in driving tumourigenesis. Conclusions: Developing a greater understanding of the influence of the microbiota on oesophago-gastric tumourigenesis may enable advances to be made in the early detection of malignancy and in the development of novel systemic therapies, leading to improved rates of survival.

## 1. Introduction

Oesophago-gastric (OG) cancer (OGC) is a disease of significant unmet clinical need, and incidence in the Western world is increasing [1]. Oesophageal squamous cell carcinoma (SCC) is the predominant subtype globally, and epidemiological studies allude to an oesophageal ‘cancer belt’, running from northern Iran, through Central Asia to North Central China; where SCC accounts for 90% of oesophageal cancers [2]. In comparison to SCC, oesophageal adenocarcinoma (AC) is the predominant subtype in Western countries including the United States, the United Kingdom and Australia [3].

### 1.1. Risk Factors for Oesophago-Gastric Cancer

Increased risk of OGC is multi-factorial, incorporating factors ranging from environmental and lifestyle to inherited genetic mutations [4]. These include smoking tobacco and consuming alcohol and refined foods with high salt content [5]. Barrett’s metaplasia is associated with an increased risk of oesophageal AC and annual risk of progression from Barrett’s to malignancy is approximately 0.11% [6]. This process is governed by numerous molecular pathways; specific genetic alterations include loss of the tumour suppressor genes p16 and p53, and an increase in cyclin D1 expression [7,8]. Additional risk factors for oesophageal AC include obesity and gastro-oesophageal reflux disease (GORD) [9]. GORD causes chronic exposure of the distal oesophagus and gastro-oesophageal junction (GOJ) to gastric and bile acids. This may result in inflammation and, eventually, Barrett’s metaplasia; transformation from stratified squamous to columnar epithelium due to direct mucosal injury from refluxate from the stomach, and indirectly by generation of reactive oxygen species (ROS), eventually leading to AC [10,11]. In comparison, smoking and alcohol cause irritation of the oesophageal epithelium, which may lead to squamous dysplasia and eventually SCC via chronic stimulation of pro-inflammatory pathways [5].

### 1.2. Initial Insights into the Oesophago-Gastric Microbiome

The upper gastrointestinal (GI) tract contains 10^14^ individual bacteria [12]. These microorganisms are involved in numerous processes including digestion, synthesis of vitamins, protection against pathogens and regulation of the host immune response [13]. A loss of physiological balance in the composition of the microbiome, which may lead to the development of diseases including cancer, is termed “dysbiosis”, and can occur in response to extrinsic and intrinsic factors [14]. Examples include antimicrobial therapy, increase in consumption of refined foods, alcohol consumption, smoking and mucosal injury to the GOJ caused by reflux of biliary and gastric acid [15,16]. Scientific evidence has confirmed that dysbiosis of the upper GI tract may be associated with the development of OGC, which may be mediated by disruption of the epithelial barrier and activation of pro-inflammatory pathways [12,15,17,18]. Chronic stimulation of these pathways may cause DNA damage to the cells comprising the OG mucosa, and may lead to tumourigenesis [19].

This review discusses the role of the microbiome as a potential driver of pro-inflammatory pathways leading to the pathogenesis of OGC, mechanisms through which this is mediated and the potential applications of gaining greater insights into the role of microbiota in OGC.

## 2. Materials and Methods

A narrative review was conducted to summarise the current evidence on the role of the microbiome in promoting OG tumourigenesis via pro-inflammatory pathways. The online scientific literature database, PubMed, was searched for relevant articles using search terms including ‘oesophago-gastric cancer’, ‘microbiome’, ‘inflammation’ and ‘tumourigenesis’. Factors identified in the scientific literature seen as major causes of dysbiosis including age, medications and GORD were examined in detail.

Key pro-inflammatory pathways, their interaction with the microbiome and mechanisms promoting tumourigenesis were drawn using the online platform, BioRender.com (Toronto, ON, Canada).

## 3. Results and Discussion

### 3.1. Profiling the Oesophago-Gastric Microbiome

The oldest reported study characterising the healthy oesophageal microbiome utilised bacterial cultures of oesophageal aspirates and ascertained 6 organisms were clearly present [20]. These included *Streptococcus viridans*, *Haemophilus influenzae*, *Neisseria catarrhalis*, *Streptococcus* group B, *Streptococcus faecalis* and *Klebsiella pneumonia* [20]. However, this represents an incomplete picture of the oesophageal microbiome, as the majority of commensal bacteria are not culturable via traditional methods. Subsequent advances in sequencing technology have led to the development of newer techniques that can provide deeper insights into the OG microbiome; identifying a larger number of genera with the addition of metagenomics to determine species. These techniques include 16S ribosomal RNA (rRNA) and shotgun sequencing.

**Figure 1:**
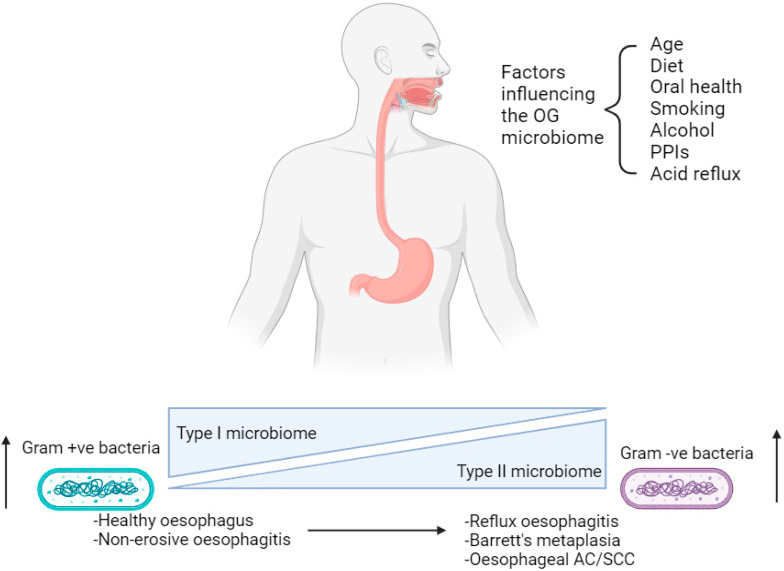
Factors affecting the oesophago-gastric microbiome in the progression from a healthy state to oesophago-gastric disease. OG; oesophago-gastric. PPIs; proton-pump inhibitors. AC; adenocarcinoma. SCC; squamous cell carcinoma. Created with BioRender.com.

The oral cavity and oesophagus both form part of the upper aerodigestive tract and arise from the common development of the primitive foregut [21]. Despite the proximity to the oral cavity, the microbiome of the upper GI tract harbours its own endemic microorganisms as well as those found in saliva, which enter the oesophagus as a consequence of the swallowing mechanism [21]. The majority of the microbiome in a healthy human oral cavity can be subdivided into six phyla: Firmicutes, Actinobacteria, Proteobacteria, Fusobacteria, Bacteroidetes and Spirochaetes [21,22]. In comparison, studies sequencing the 16S rRNA gene have identified a distinct OG microbiome in which clear differences are observed at a genus level, although there is a similarity in the phyla that are present [23]. An example of such a study was performed by Pei et al., in which ninety-five species-level operational taxonomic units (OTUs) have been detected, belonging to six phyla; Firmicutes, Bacteroidetes, Actinobacteria, Proteobacteria, Fusobacteria and Saccharibacteria (TM7) within the healthy oesophagus. Clustering analyses performed on microbial data have identified that two sub-types of microbiota exist in upper GI disease [23]. A type I microbiome is associated with a healthy oesophagus and contains a preponderance of gram-positive bacteria, especially *Streptococcus* species (spp.); whereas a type II microbiome has a larger proportion of gram-negative anaerobic organisms including *Bacteroidetes*, *Proteobacteria*, *Fusobacteria* and *Spirochaetes* [23,24]. The latter is correlated with reflux oesophagitis, Barrett’s metaplasia and oesophageal AC (Figure 1) [25,26,27]. However, just four adult human subjects were sampled in Pei et al.’s study, resulting in a need for larger studies involving a greater number of subjects in order to make more definitive conclusions [23].

### 3.2. Factors Influencing the Oesophago-Gastric Microbiome

The composition of the OG microbiome may change in response to age, medication and diet, leading to dysbiosis (Figure 1) [18]. This phenomenon is associated with OG tumourigenesis via disruption of the epithelial barrier causing chronic inflammation and inducing DNA damage [16,17]. Numerous studies in the scientific literature characterise the OG microbiome either in different disease states or in the context of different environmental factors, including medications and diet. This reiterates the influence these variables have on the presence of bacteria within the upper GI tract.

Increasing age is associated with a rise in the pH of gastric acid [28]. Acid reflux provides a vehicle through which this modulates the oesophageal microbiome [28]. A decrease in the acidity of gastric acid and the degenerative effect of aging on oesophageal motor function causes a change in the conditions the oesophagus is exposed to, stimulating further OG dysbiosis [29]. A study by Deshpande et al. used 16S and shotgun sequencing methods to identify specific changes in microbial signatures that occur as a result of age in 106 adult subjects [30]. These include an increase in prevalence of *Streptococcus* spp., especially *S. parasanguinis* and a decrease in gram-negative *Prevotella* spp., including *Prevotella melaninogenica* [30]. The subjects in this study were aged between 49 and 65.1 years; however, peak incidence of oesophageal cancers tends to be later in life, at approximately 75–80 years. Recruiting subjects within this age range may yield a more accurate depiction of the microbiome in oesophageal cancer. This would enable comparisons with the microbiome in oesophagitis, GORD and Barrett’s metaplasia to be made.

Non-erosive GORD is associated with increased expression of Proteobacteria and Bacteroidetes, and a decrease in Fusobacteria [16]. In comparison, reflux oesophagitis is characterised by a decrease in Firmicutes and increase in Fusobacteria and Proteobacteria [16]. This latter change in bacterial prevalence persists in the progression to Barrett’s metaplasia and oesophageal AC. Understanding the factors and mechanistic pathways that promote dysbiosis along this spectrum from benign to malignant OG disease may lead to the discovery of biomarkers that could be used to improve early detection rates of OGC and, therefore, potentially improve survival.

Changes in the OG microbiome correlate with the development of oesophageal diseases; in particular, GORD and OG malignancy. Medications taken to manage GORD include proton-pump inhibitors (PPIs). Specifically, PPIs are weak bases with a pK_a_ of 4.0–5.0. They accumulate in the secretory canaliculi of gastric parietal cells and bind to the H^+^,K^+^-ATPase antiporter pumps, rendering these pumps inactive, inhibiting secretion of gastric acid and, thereby, increasing the pH of gastric content [31]. This leads to reduced corrosion of the mucosa of the stomach and GOJ during reflux [31]. A study of 34 subjects with Barrett’s metaplasia, oesophagitis or a normal distal oesophagus using 16S rRNA gene sequencing identified increases in distal oesophageal *Lachnospiraceae*, *Comamonadaceae* and *Clostridial* families in subjects taking PPI therapy [32]. Furthermore, *Methylobacteriaceae*, which were increased in gastric aspirates among subjects with Barrett’s metaplasia or oesophagitis before PPI therapy were significantly reduced after treatment and are often found in inflamed mucosa [33]. It may be hypothesised that the changes in the digestive ecosystem cultivated by PPIs may lead to conditions in which certain bacterial families thrive, resulting in changes in the microbiota. In Amir et al.’s study, patients referred for investigation of heartburn were studied. However, those that were taking antibiotic or acid-suppressing therapy in the two months prior to sampling were excluded [32]. The treatment of heartburn symptoms may involve medications including liquid sodium alginate, which also contains calcium carbonate and sodium bicarbonate, and other factors including limiting intake of foods high in ascorbic acid or citric acid and avoiding eating late at night. This study does not adjust for these factors in the clinical profiling of the recruited subjects, which could also affect the microbiome of the gastric fluid and lower oesophageal mucosa when sampled.

Environmental and lifestyle factors; in particular, diet, have a major influence on the OG microbiome [18]. According to the literature, diet is associated with several oesophageal diseases, including OGC. Specifically, high consumption of red and processed meats and alcohol are associated with an increased risk of malignancy; whereas whole grains, fruits, leafy green vegetables, and foods high in zinc and calcium are associated with a lower risk [34,35]. Red and processed meats contain high proportions of compounds including heterocyclic amines, polycyclic aromatic hydrocarbons and N-nitroso compounds which exert a mutagenic effect on the OG mucosa [36]. Increase in dietary fibre is associated with a distinct oesophageal microbiome, as evidenced by a 16S high-throughput sequencing study performed by Nobel et al. [37]. Statistically significant increases in the relative abundance of *Firmicutes* (*p* = 0.04) and a reduction of Gram-negative bacteria including *Prevotella*, *Neisseria* and *Eikenalla* (*p* = 0.03) were associated with an increase in fibre intake [37].

A risk factor common to a range of both benign and malignant diseases is tobacco smoke. Long-term cigarette smokers (smoking for ≥40 years) are twice as likely to develop OG cancer compared to non-smokers [38]. Noxious substances in cigarette smoke, including polycyclic aromatic hydrocarbons and N-nitrosamines directly cause chronic irritation and subsequent tissue damage to the oesophageal epithelium [39]. This may lead to squamous dysplasia and eventually oesophageal SCC through the production of ROS [39]. Pairing chronic smoking with alcohol consumption has a synergistic effect, which perpetuates the risk of oesophageal SCC further [40]. As well as exerting a direct carcinogenic effect on oesophageal mucosa, smoking can cause dysbiosis of the oral microbiota, which is associated with oesophageal cancer [41]. Although the literature suggests that the microbiota of the oral cavity is distinctly different to the oesophagus, smoking causes local immunosuppression and biofilm formation, which may lead to colonization by harmful bacteria and distal tumourigenesis within the oesophagus [18]. Furthermore, dysbiosis can cause disruption of the local epithelial barrier, chronic inflammation and carcinogenesis through DNA damage [17].

As highlighted in this review, many studies have been carried out to profile the differences in the OG microbiome in the context of different influential factors including diet, medications and advanced age. There are local and systemic processes that are involved in driving the changes in microbial presence [19].

### 3.3. Pro-Inflammatory Pathways in Oesophago-Gastric Tumourigenesis

**Figure 2:**
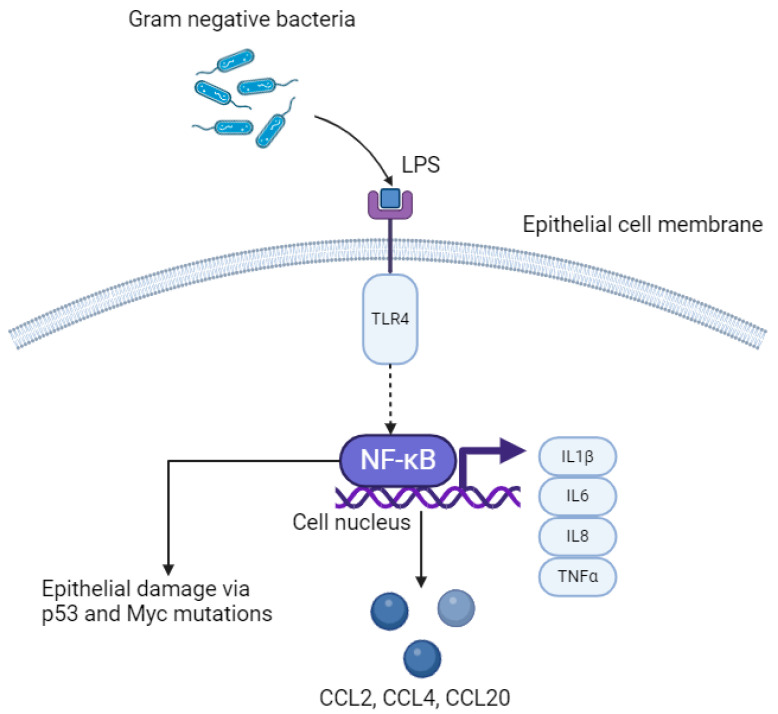
LPS–TLR4–NFκB pathway in oesophageal epithelial cells. LPS; lipopolysaccharide. TLR4; Toll-like receptor 4. NFκB; nuclear factor kappa B. IL; interleukin. TNFα; tumour necrosis factor alpha. Created with BioRender.com.

**Figure 3:**
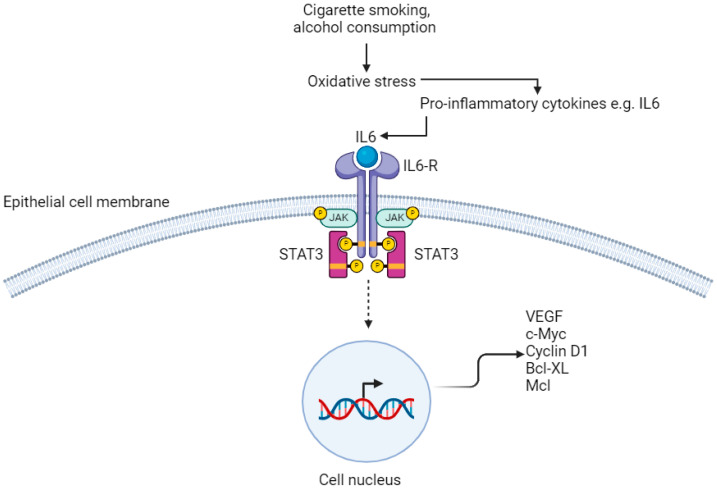
IL6/STAT3 pathway. IL6-R; Interleukin-6 Receptor. JAK; janus kinase. STAT3; signal transducer and activator of transcription VEGF; vascular endothelial growth factor. Created with BioRender.com.

Oesophago-gastric cancer is associated with a bacterial profile that activates the innate immune system, and initially triggers metaplasia and dysplasia [18]. Downstream stimulation of cytokine-mediated pro-inflammatory pathways following microbial perturbation, as a result of phenomena including gastro-oesophageal reflux and carcinogens including tobacco smoke, are vital in inducing proliferation of OGC [16]. This may be mediated by immune cells and the chronic release of inflammatory mediators including transcription factors; tumour necrosis factor alpha (TNFα) and chemokines; CCL20, CCL4, CCL2 [18,19,42,43]. The importance of inflammation in giving rise to tumourigenesis has been demonstrated in both in vitro studies and in animal models [43]. Specifically, bacteria colonise the mucosa and interact with epithelial cells via cell surface receptors [44,45]. Fu et al. demonstrated this using *Streptococcus anginosus* in a murine model, through its interactions with the TMPC–ANXA2–MAPK axis, culminating in gastric inflammation, followed by atrophy and then tumourigenesis [46]. OGC has been demonstrated to have a major pro-inflammatory component; activation of the nuclear factor kappa B (NF–κB) (Figure 2) and interleukin-6/signal transducer and activator of transcription 3 (IL6/STAT3) (Figure 3) pathways lead to transcription of genes that lead to tumour growth [47].

### 3.4. LPS–TLR4–NFκB Pathway

A greater proportion of gram-negative to gram-positive bacteria in the oesophageal mucosa is termed a type II microbiome and has been identified in Barrett’s metaplasia and oesophageal AC [24]. The cell walls of bacteria are mostly composed of peptidoglycan, and lipopolysaccharide (LPS) is an endotoxin forming a component of the outer cell wall in gram-negative organisms [48]. LPS is a pathogen-associated molecular pattern (PAMP) which activates the innate immune response by acting as a natural ligand to Toll-like receptor 4 (TLR4) on epithelial cells (Figure 2) [49]. Proteins including cluster of differentiation 14 (CD14), which are expressed by macrophages and dendritic cells as well as GI epithelial cells bind to LPS first, forming a ternary complex called LPS-binding protein:CD14. This enhances binding of LPS to TLR4. Other natural ligands to TLR4 include myeloid differentiation protein 2 (MD2) and can increase binding of LPS to TLR4. Within the context of OG cancer, binding of LPS to TLR4 causes activation of the NF–κB pathway, which leads to inflammation-related carcinogenesis via an increase in pro-inflammatory cytokines, including interleukins (IL) 1β, IL6, IL8 and TNFα, which are released directly from epithelial cells [50]. Continuous activation of this pathway leads to chronic inflammation, and ultimately may induce tumourigenesis. Specific bacterial species that activate the LPS–TLR4–NFκB pathway, that has been isolated in oesophageal tumour tissue, includes *Fusobacterium nucleatum* [51]. This species in particular is associated with rapid tumour progression through activation of chemokines including CCL20 [18]. Furthermore, increased activation of NFκB results in interruption to the epithelium, leading to DNA damage via p53 and Myc mutations, promoting OG carcinogenesis [52].

A secondary effect of activation of the LPS–TLR4–NFκB pathway as a consequence of greater abundance of gram-negative bacteria is the upregulation of genes encoding the pro-inflammatory enzyme, cyclooxygenase-2 (COX-2) via the mitogen-activated protein kinase (MAPK) pathway [18]. This is associated with an increase in progression from Barrett’s metaplasia to oesophageal AC [53,54]. Furthermore, increased TLR4 binding results in greater activation of inducible nitric oxide synthase (iNOS), which has been demonstrated to relax the lower oesophageal sphincter in mice treated with LPS, through phosphorylation of MAPK [55]. The resultant effect of further exposure of the GOJ to gastric and biliary acid suggests a positive feedback loop may exist, perpetuating progression to oesophageal AC from Barrett’s metaplasia.

### 3.5. Inflammasome-Mediated Oesophago-Gastric Tumourigenesis

Further studies have highlighted the role of inflammasomes mediated by gram-negative bacteria in OG carcinogenesis [49]. Inflammasomes are cytoplasmic protein complexes composed of enzymes including caspase-1 and caspase-5 [56]. These enzymes cleave interleukins from their precursor to activated forms; pro-IL1β and pro-IL18 to IL1β and IL18, respectively; a key step in apoptosis [57]. LPS, a PAMP, binds pattern-recognition receptors (PRRs) on the nod-like receptor protein 3 (NLRP3) inflammasome, which mediates the secretion of pro-IL1β and pro-IL18, and activation of caspase-1. Activation of caspase-1, IL1β and IL18 causes the release of ROS, which damages oesophageal DNA and promotes progression to oesophageal AC from Barrett’s metaplasia [49].

The tumorigenic effect of an increase in production of pro-inflammatory cytokines via the LPS–TLR4–NFκB pathway is mediated by infiltration of immune cells, evasion of apoptosis, immune suppression and stimulation of angiogenesis [58]. These cytokines are chemotactic and can modulate the tumour microenvironment by concurrently promoting both infiltration of leucocytes into the tumour and tumour cell growth [58,59,60]. Examples of chemokines overexpressed in oesophageal tumours include IL8, CXCL1 and CXCL3 [61].

### 3.6. IL6–STAT3 Pathway

Chronic inflammation of the OG mucosa from exposure to carcinogens including cigarette smoke and excess alcohol consumption results in activation of the IL6–STAT3 signalling pathway, which promotes proliferation of oesophageal SCC [18,62,63]. Receptors on the membrane of oesophageal squamous epithelial cells have both extra-cellular and intra-cellular components. Binding of IL6 results in oligomerisation and activation of receptors, which causes their intra-cellular components to bind to each other [62,64]. This leads to activation of receptor-coupled tyrosine kinases by cross-phosphorylation. These tyrosine kinases are Janus kinase (JAK) family members and their phosphorylation results in binding of STAT3 molecules to the cell membrane via the SH2 domain [65]. JAK phosphorylates the tyrosine residue of STAT3, causing it to separate from the receptor, transfer to the nucleus and bind to DNA, affecting gene transcription (Figure 3) [66]. In particular, STAT3 upregulates the expression of genes that are required for angiogenesis; vascular endothelial growth factor (VEGF), uncontrolled proliferation; c-Myc and cyclin D1, and anti-apoptotic genes; Bcl-XL and Mcl [64,67,68,69,70].

A prospective, national, nested case-control study by Peters et al. identified that increased abundance of *Porphyromonas gingivalis* in the oral cavity was associated with greater risk of oesophageal SCC [41]. This is a gram-negative pathogen associated with periodontitis and other oral diseases but can induce chronic inflammation of the oesophagus and proliferation of oesophageal SCC via both the NFκB and IL6–STAT3 pathways [71,72,73]. In comparison, a study by Chen et al. demonstrated that IL6 expression increased in the supernatant of cultured oesophageal cancer cells infected with *Porphyromonas gingivalis*, and that IL6 levels in primary human oesophageal cancer are positively associated with the presence of this organism [73]. Of note, Peters et al. analysed oral mouthwash samples from 316 subjects, adjusting for smoking and alcohol consumption as well as frequency of fruit and vegetable consumption [41]. Despite identifying an organism associated with periodontitis that had greater abundance among patients who developed oesophageal SCC, an objective assessment of these patients’ oral health was not made, e.g., by questionnaires. Therefore, future studies could include this when investigating potential associations between oral health and incidence of oesophageal cancer.

Both LPS–TLR4–NFκB and IL6-STAT3 are pro-inflammatory pathways that promote tumourigenesis following disruption of the relationship between commensal bacteria and epithelial cells. The literature suggests that these pathways also interact with each other and work synergistically in a positive feedback loop, with similar downstream effects [52,74,75]. Of note, STAT3 interacts with NFκB at multiple steps along their pathways and is activated by IL6, a pro-inflammatory cytokine that is another product of LPS–TLR4–NFκB pathway activation. Both STAT3 and NFκB are transcription factors that regulate several genes that are key in STAT3 activation and pro-tumourigenic inflammation [75]. Furthermore, inhibition of one of these pathways results in increased activation of the other, which can lead to the adaptation and greater resilience of cancer cells, contributing to further disease progression [76].

### 3.7. The Role of Immune Modulation in Oesophago-Gastric Cancer

Evasion of the host’s immune system is needed for tumour survival and disease progression. The tumour microenvironment in OG cancer typically consists of CD8+ T cells that are angiostatic, anti-tumourigenic and stimulate the immune system against tumour cells that are pro-fibrotic and angiogenic [52]. Evidence has demonstrated that OG tumour cells can suppress the anti-tumour immune response by recruiting immune cells or expressing inhibitory molecular factors [52].

Examples of cells include myeloid-derived suppressor cells (MDSCs), regulatory T cells and Th17 cells. MDSCs are involved in fibroblast activation and angiogenesis, and are triggered by pro-inflammatory cytokines including IL1β, IL6 and vascular endothelial growth factor (VEGF). These properties enable them to promote tumour growth. Concurrently, they can inhibit the anti-tumour immune responses mediated by T cells through direct inhibition of T cells and natural killer cells [52]. Regulatory T cells are recruited by chemokines CCL17 and CCL22, which are released by both tumour cells and macrophages in oesophageal SCC [52,77]. Greater infiltration of regulatory T cells is associated with greater tumour invasion and disease severity. Th17 cells promote tumour growth and angiogenesis through IL17 and IL22, via STAT3 activation [78].

Developing a greater understanding of the immune-related mechanisms of OGC has led to the development of novel therapies associated with greater overall survival in advanced disease [74]. One group of novel agents are immune checkpoint inhibitors. Pembrolizumab is one particular example. This is an IgG4 monoclonal antibody that binds to programmed cell death protein-1 (PD-1) receptors on T cells, preventing programmed cell death ligands (PD-L1) on cancer cells binding instead [79]. This stops inhibition of the host’s immune response, enabling the host to combat the cancer cells. An interim analysis of the KEYNOTE-585 trial showed that neoadjuvant and adjuvant pembrolizumab and chemotherapy in locally advanced, resectable gastric or gastro-oesophageal cancer is associated with a greater pathological complete response rate [79]. This reiterates the importance of further research into the immune landscape of OGC in terms of identifying new systemic treatments that can be used in conjunction with radical surgery to improve long-term survival outcomes.

### 3.8. Pro-Tumourigenic Pathways Induced by Diet

The influence of dietary choices on development of OGC has been studied extensively; specific food types are associated with greater risk of gastric cancer, in particular. Far Eastern countries including Japan and South Korea have a higher incidence of gastric cancer than Western countries, and their diets typically contain a higher proportion of salt-preserved foods including fish and pickled vegetables [80]. It has been highlighted in pre-clinical and epidemiological studies that consumption of smoked and salt-preserved foods is a risk factor associated with tumourigenesis via the effect of nitrates [81,82,83]. Nitrates are absorbed from food in the small intestine and approximately 25% is re-secreted into the saliva. Bacteria on the tongue including *E. coli* reduce this to nitrite and the swallowing mechanism results in nitrite entering the stomach. Gastric juices containing hydrochloric acid and ascorbic acid convert nitrite into nitric oxides, which damage gastric mucosa and induce DNA mutations [82,83,84]. Areas of injury to the gastric mucosa become sites of inflammation, resulting in local release of free radicals including hydroxyl ions (OH^+^) and nitric oxides (NO_2_^−^), as well as ROS during tissue repair [85,86]. This causes oxidative DNA damage, and reaction of nitric oxides with oxygen to form dinitrogen trioxide (N_2_O_3_) [86]. Both mechanisms stimulate mutations through the deamination of DNA and formation of *N*-nitrosocompounds [87]. A study by Iijima et al. used a nitric oxide sensor, demonstrating that the highest concentration of nitric oxide within the stomach is found at the GOJ and cardia [87]. This reiterates the role of nitrates in the pathogenesis of certain cancers of the GOJ and gastric cardia. Consumption of red meat has also been linked to the development of gastric cancer via the endogenous production of *N*-nitrosocompounds, mediated by its high concentration of haem, which is significantly greater than in white meat [88].

### 3.9. Barrett’s Metaplasia and Progression to Oesophageal and Gastro-Oesophageal Junction Adenocarcinoma

Pathogenesis of Barrett’s Oesophagus occurs over a two-step process. The first is transformation from oesophageal squamous mucosa to columnar epithelium, termed ‘metaplasia.’ This is followed by development of goblet cells within the columnar epithelium, which is representative of intestinal metaplasia [10,11]. This process can be triggered by gastro-oesophageal reflux and subsequent exposure of the oesophagus to gastric acid, although the exact molecular mechanism by which this occurs is unknown [10,11]. However, it is hypothesised that exposure of oesophageal squamous mucosa to gastric acid produces dilated intercellular spaces that enable molecules up to 20 kDa in size to permeate down to the stem cells in the basal layer and induce a transformation resulting in production of columnar epithelial cells over squamous cells [89].

Barrett’s metaplasia is a precursor to AC of the oesophagus and GOJ [90,91]. Risk factors of Barrett’s include GORD, obesity and smoking. Of these, GORD and subsequent inflammation of the oesophagus is most heavily linked to Barret’s metaplasia. However, this is reported to be promoted further by changes in the microbiome; notably, an increase in the prevalence of gram-negative organisms, which in conjunction with risk factors including GORD, may promote Barrett’s metaplasia and progression to cancer [15,90]. Despite this, the exact interplay between dysbiosis and inflammation leading to pathogenesis of Barrett’s, and subsequent progression to AC is not fully characterised in the literature, highlighting a key area for future research.

Low-volume cross-sectional studies have identified that gram-negative bacteria including *Fusobacterium nucleatum* and *Eschericia coli* (*E. coli*) are of higher prevalence than gram-positive organisms in Barrett’s, but causality is yet to be confirmed [90]. As described in Figure 2, LPS produced by gram-negative organisms triggers the innate immune response, stimulating TLR4 and activating the NF–κB pathway, which results in an increase in IL1β, IL6 and TNF [15,18]. This increase in pro-inflammatory cytokines is representative of the metaplasia-dysplasia sequence that leads to oesophageal adenocarcinoma [15,92]. Specifically, *Fusobacterium nucleatum* binds to epithelial cadherin (*E. cadherin*) and activates β-catenin, which leads to TLR4 activation, NF–κB signalling and production of microRNA-21 in tumourigenesis (miR-21) [93].

### 3.10. Systemic Effect of Dysbiosis in Oesophago-Gastric Tumourigenesis

The scientific literature also suggests that dysbiosis promotes OG tumourigenesis through a systemic response, which may also contribute to metastasis [70]. Following dysbiosis, alterations occur, including inflammation, activation of an immune response and production of genotoxins. Examples of these alterations include the production of bacterial metabolites, which are recognised by immune cells via PAMPs and signal via the myeloid differentiation primary response protein (MyD88)-dependent pathway. Furthermore, PAMPs, including LPS, activate dendritic cells which stimulate the production of CD4^+^ regulatory T cells, T Helper 17 (Th17) cells and CD8^+^ T cells [70,94]. This results in the production of interferons that can drive the metastatic spread of malignancy [94]. Overall, chronic inflammation is the main process most frequently cited in the literature when explaining tumourigenesis in OGC following dysbiosis [18]. Alongside activation of pro-inflammatory pathways, bacteria may use genotoxins to cause direct structural damage to DNA, resulting in cell mutation and carcinogenesis [95]. Examples include cytolethal distending toxin (CDT) and cytotoxin-associated gene A (CagA), which are produced by *Escherichia coli*, *Actinobacillus actinomycetemcomitans*, *Campylobacter* spp. and *Helicobacter pylori* (*H. pylori*) [19,70,96]. Genotoxins invade cells and stimulate production of ROS which enter the nucleus to induce DNA damage and ultimately cell mutation [19].

Although the mode of transmission of *H. pylori* is not clearly outlined in the literature, evidence has alluded to a faecal–oral or oral–oral route through water or food consumption, as a potential cause [97]. Cellular mechanisms by which CDT, which is transported by *H. pylori*, stimulate oncogenesis include disruption of DNA repair and dysregulation of cell cycle progression [98]. First, CDT enters cells by binding to holotoxin which contains CdtB, enabling it to cross the cell membrane and enter the nucleus [99].

CagA is an effector protein secreted by the Type IV secretion system directly into the cytoplasm of gastric epithelia [100,101]. For this to occur, surface adhesins on *H. pylori* interact with receptors on host gastric epithelial cells derived from carcinoembryonic antigen-related cell adhesion molecules [101]. CagA stimulates malignant transformation of epithelial cells by promoting cellular phenotypes typically associated with cancer, including proliferation, resistance to cell death, genomic instability and evasion of tumour suppression [102]. Consequently, individuals infected with CagA-positive *H. pylori* are more likely to develop gastritis, ulceration and *H. pylori*-associated gastric cancer.

A further protein found in all human strains of *H. pylori* is vacuolating cytotoxin A (VacA). VacA enables *H. pylori* to colonise and survive in the stomach by resisting host immune defences, causing persistent infection [103]. Specifically, VacA inhibits the activation of T cells and the induction of regulatory T cells, inhibits immune cell proliferation and stimulates mast cells to produce pro-inflammatory cytokines [104]. This contributes to chronic inflammation and subsequent ulceration of the gastric mucosa [103]. The m1 and s1 allelic forms of VacA are associated with increased risk of *H. pylori*-associated gastritis, subsequent peptic ulcer disease and eventually gastric cancer [96,105].

### 3.11. Role of Short-Chain Fatty Acids in Oesophago-Gastric Tumourigenesis

**Figure 4:**
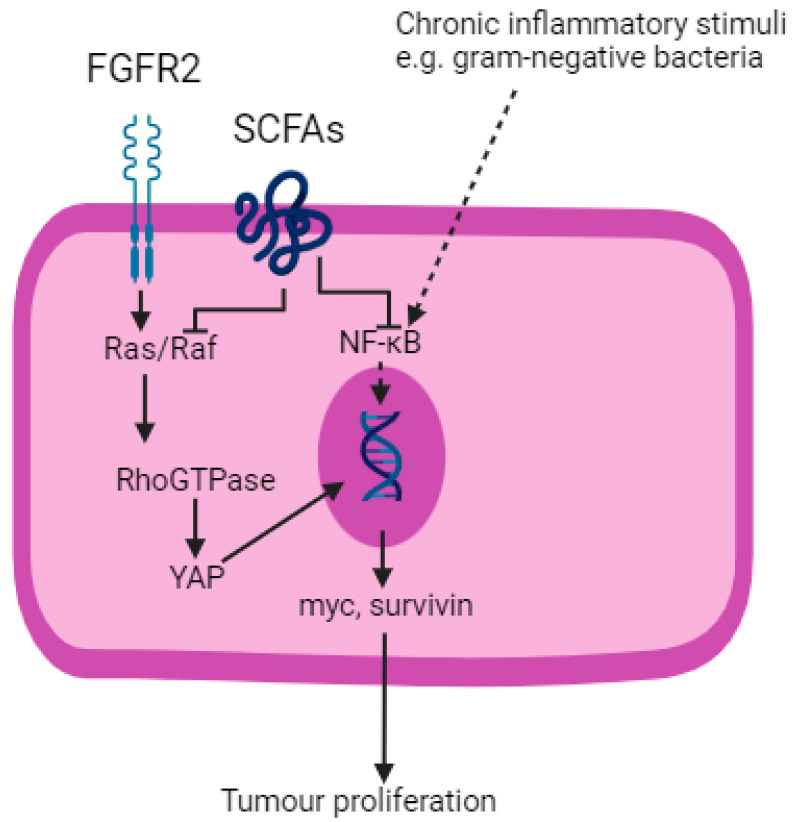
SCFAs inhibit Ras/Raf and NF–κB pathways. FGFR2; fibroblast growth factor receptor 2. SCFAs; short-chain fatty acids. YAP; yes-associated protein. Created with BioRender.com.

A key pathway identified in the scientific literature that may lead to tumourigenesis is mediated by a decrease in short-chain fatty acids (SCFAs) in the GI tract (Figure 4) [106]. The microbiome of the GI tract produces three types of SCFAs: butyrate, propionate and acetate, as by-products of the fermentation of dietary fibre by gram-negative commensal bacteria including the Prevotella and Bacteroides genera [106]. SCFAs are carboxylic acids that are between one and six carbon atoms in length. Their functions include regulation of mucosal immunity, immunomodulation of macrophages and anti-inflammatory effects on lymphocytes [106,107]. A fall in the levels of SCFAs in the GI tract due to a reduction in the intake of dietary fibre is a hallmark of dysbiosis and has been identified in patients with solid organ malignancies of the oesophagus and stomach [108].

SCFAs are ligands to G-protein coupled receptors, enabling them to enter cell nuclei and exert anti-tumourigenic effects including histone deacetylase inhibition [109]. Furthermore, SCFAs promote the production of anti-microbial peptides including α-defensins in the GI tract, which prevent bacterial penetration through the epithelium via PAMPs including LPS [106]. This may otherwise trigger a chronic inflammatory process and lead to tumourigenesis [110].

Blockade of the Ras/Raf and NF–κB pathways are two mechanisms by which SCFAs exert their anti-tumourigenic effects [109]. Fibroblast growth factor receptor 2 (FGFR2), a tyrosine kinase receptor, activates the Ras/Raf pathway, which is a component of the Hippo signalling pathway in the development of OGC [111]. Activation of the Ras/Raf pathway leads to downstream activation of Yes-associated protein (YAP) and which results in the transcription of oncogenes including myc and survivin [109]. SCFAs block Ras signalling and trigger apoptosis.

As described, the NF–κB pathway is ubiquitous to many solid organ malignancies that arise from chronic inflammation, including OGC. SCFAs downregulate pro-inflammatory cytokines produced as a result of NF–κB pathway activation, replacing them with IL-10 and Transforming Growth Factor beta (TGFβ), which are anti-inflammatory [112]. Butyrate also inhibits the STAT3 pathway and thereby reduces the production of bcl-2, c-myc and cyclin D1, leading to reduced cell proliferation and increased apoptosis [113].

SCFAs are key metabolites produced by gram-negative bacteria within the GI tract. Their immunomodulatory effects play a significant role in mediating OG tumourigenesis due to their involvement in several major pro-inflammatory pathways. This represents another example of the role of the microbiome in tumourigenesis. This reiterates the importance of examining this in further detail to elucidate potential markers of early disease that can be used to improve rates of early detection and long-term survival.

### 3.12. Therapeutic Application of Insights into Microbiome-Mediated Oesophago-Gastric Tumourigenesis

Potential advances in early diagnosis may be associated with greater survival in OGC. However, OGC is a systemic disease and there is a significant need for more effective therapies. The microbiome is a complex, dynamic entity heavily influenced by factors including diet, medications and age. As well as having a role in tumourigenesis, which is mediated through chronic inflammation, certain microorganisms may exhibit anti-cancer properties creating potential future therapeutic options. These have been identified in other solid organ malignancies, including pancreatic and colon cancers.

The organism *Clostridium perfringens* enterotoxin (CPE) binds to transmembrane tight junction proteins claudin-3 and claudin-4, expressed in colon cancer [114]. This interaction leads to loss of osmotic equilibrium between intracellular and extracellular fluids, resulting in cell death [115]. Where toxins including CagA, VacA and CDT have pro-tumourigenic properties, *Clostridium perfringens*, *Salmonella typhimurium* and *Pseudomonas aeruginosa* generate toxins that are hazardous to healthy cells [115]. This makes them potentially modifiable in a way that enables cancer cells to be targeted by creating new systemic therapies or augmenting existing treatments [114,115].

The idea of treating cancer by introducing microorganisms into the tumour to induce tumour regression has been previously investigated but in limited detail. Oncolytic viruses have undergone various stages of clinical trials but little work has been carried out on the modification of bacteria for treatment [116]. Several microorganisms of interest are expressed in the GI tracts of patients with colorectal cancers and have been identified to have cancer-inhibiting effects in animal models [115]. Examples include *Lactobacillus* spp. and *Bifidobacterium*. In the case of the former, *L. rhamnosus* is reported to stimulate a type I interferon response via the cGAS/STING signal transduction pathway, improving the response to immune checkpoint inhibitors [117]. *Bifidobacterium* induces the maturation of dendritic cells, activating IFNα and IFNβ signalling, as well as stimulating CD8+ T cells and thereby promoting the anti-tumour response [118].

Further work is therefore required to understand the potential role of the microbiome in the treatment of OGC; for example, whether it may be used to increase the efficacy of chemotherapy to destroy cancer cells or block its ability to modify the immune response.

Another potential avenue for further research could be treatment to eliminate certain microorganisms that are strongly associated with specific cancers. This has already been established with treatment of *H. pylori* in reducing the risk of gastric cancer but could be extended to other bacteria. Peters et al. demonstrated increased expression of *P. gingivalis* in the mouthwash of patients with oesophageal SCC [41]. This bacterium is also closely associated with periodontitis. Treatment of patients with antibiotics specifically targeting these bacteria could potentially help reduce the risk of the development of cancer. However, given the diversity of the microbiome and lack of highly specific antibiotic treatments, there is a potential risk of concurrently destroying commensal bacteria that, e.g., are involved in immunity, digestion and metabolism.

## 4. Limitations

Significant research has been carried out attempting to characterise the OG microbiome and understand the mechanistic role it may play in tumourigenesis. However, there is considerable heterogeneity between the studies reported in this review. As described, many confounding factors influence the OG microbiome and adjusting for them all is challenging and consequently requires a high number of subjects in order to potentially draw valid conclusions. An additional confounder includes geography; where the subjects have been recruited from and the impact of local environmental conditions on the microbiome [119]. The studies discussed in this review have been carried out all over the world and therefore, to a certain extent, the microbial profiles of the subjects reported has been influenced by the local environment including water, food and atmospheric composition [119].

The OG microbiome is a dynamic entity and a lot of current evidence is anecdotal involving a low number of subjects or is cross-sectional in nature that may not account for many of the confounders as previously described. This limits the applicability of the conclusions that can be drawn from these studies. Furthermore, new sequencing methods including 16S rRNA and shotgun sequencing have been used to characterise the microbiome. Other examples include whole-exome sequencing and other next-generation sequencing techniques, which may yield further insights into the microbiome. However, these are associated with significant costs, which may explain why many of the current studies are low-powered or do not provide deeper insights into the composition of the microbiome.

## 5. Conclusions

This review describes the influence of the microbiome on OG tumourigenesis and explains how various factors, ranging from diet to oral health and medications, may contribute to dysbiosis. Inflammation is a major driver of OGC and there are many components of pro-inflammatory pathways that contribute to development of OGC. Novel methods including 16S rRNA sequencing have enabled a greater understanding of the microbiome to be gained, but metagenomic studies are needed to gain deeper insights into the microbiome at a species level alongside analysis of bacterial traits including virulence, adherence or antimicrobial resistance. Although numerous observational studies have attempted to profile the microbiome in different cohorts, there is significant heterogeneity both between individual subjects and between populations on national and international scales. This is because of the multitude of genetic and environmental factors that influence the microbiome. This suggests that larger-volume studies accounting for confounding factors including diet and oral health are required. Furthermore, this could involve healthy controls, subjects with benign upper GI diseases as well as Barrett’s metaplasia and OGC in order to profile the OG microbiome during tumourigenesis. Given that the microbiome is a constantly evolving entity, there is a need for longitudinal studies which could involve sampling subjects at multiple time points. This could enable more subtle changes that may be associated with the development of diseases to be elicited.

To complement these analyses and gain greater understanding of the microbiome’s structure and functions within the body, proteomic and metabolomic studies could be performed. This would enable the proteins within the cells and metabolites that are produced as a product of cellular functions to be elicited. Consequently, the role of the microbiome in the development of OGC could be determined and investigated further to identify markers of early disease and potential therapeutic targets.

Finally, although specific bacterial species implicated in OGC have been identified, further understanding of the mechanistic processes that drive OG tumourigenesis from healthy oesophageal mucosa in vivo are needed. The translational application of this could include the development of risk stratification tests that may be used to identify patients with possible early malignancy who may undergo endoscopy and biopsy for diagnostic confirmation. Early diagnosis is associated with greater likelihood of treatment with curative intent, and may improve rates of survival in OGC.
